# Optimization of protein extraction from skin tape strips for biomarker assessment

**DOI:** 10.1038/s41598-026-44234-9

**Published:** 2026-04-11

**Authors:** Tiana Stanisic, Caroline Meyer Olesen, Maria Oberländer Christensen, Beatrice Dyring-Andersen, Marianne Bengtson Løvendorf, Jop Vreeken, Florentine de Boer, Sanja Kezic, Martin Kongsbak-Wismann

**Affiliations:** 1 LEO Foundation Skin Immunology Research Center, Faculty of Health and Medical Sciences, University of Copenhagen, Copenhagen, Denmark; 2 Department of Dermatology, Zealand University Hospital, Roskilde, Denmark; 3 Department of Dermato-venereology, Bispebjerg Hospital, Copenhagen, Denmark; 4 LEO Pharma A/S, Ballerup, Denmark; 5 Department of Dermatology and Allergy, Herlev and Gentofte Hospital, Copenhagen University Hospitals, Copenhagen, Denmark; 6 Department of Public and Occupational Health, Amsterdam Public Health Research Institute, Amsterdam University Medical Center, University of Amsterdam, Amsterdam, The Netherlands

**Keywords:** Skin, Tape strips, Chronic hand eczema, Protein, Biomarker, Extraction, Biochemistry, Biological techniques, Biomarkers, Diseases, Medical research

## Abstract

Stratum corneum tape-stripping is a non-invasive alternative to biopsies for biomarker analysis in inflammatory skin diseases, but low-abundance protein detection is challenging. High extraction yield is therefore crucial, yet extraction protocols vary. Because total protein collected on the tapes is commonly used for biomarker normalization, factors influencing its measurement can impact data interpretation. We sought to investigate how extraction conditions such as buffer type, volume, and sonication time affect both total protein and cytokine biomarker yield. Sequential tape strips were collected from lesional skin of 19 patients with chronic hand eczema and matched skin sites in 13 healthy controls. Tapes were cut for equal allocation across experimental conditions. Total protein was measured by the Micro BCA assay and biomarkers by the ELISA and MSD immunoassays, with blank tapes and buffers as controls. T-PER buffer with protease inhibitor CAA yielded higher cytokine levels than PBS or PBS-Tween ± CAA, but also increased BCA assay background levels. Longer sonication and smaller extraction volumes exacerbated background without improving cytokine yield. Hence, normalization of biomarker levels may be confounded by these methodological factors. Considering the buffer, its volume, sonication time, and the inclusion of negative controls is therefore instrumental when designing studies.

**Supplementary Information:**

The online version contains supplementary material available at 10.1038/s41598-026-44234-9.

## Introduction

Tape stripping is a method of acquiring biological material from the skin, with applications in research on the skin barrier, disease biomarkers in inflammatory skin diseases such as chronic hand eczema (CHE), and treatment response monitoring. Its non-invasive nature facilitates study participation compared to studies using skin biopsies, especially in pediatric cohorts or when sampling more sensitive sites like the hands^1,2^. This facilitates the inclusion of larger sample sizes and thus provides improved resolution of biomarker signatures in heterogeneous inflammatory diseases. However, achieving detectable levels of low-abundance biomarkers from tape strips can be challenging, and protocol optimization is therefore warranted^3–5^.

Many protocols for the extraction of intact proteins from tape strips use phosphate-buffered saline (PBS) with a low concentration of Tween-20, and sonication for ≥ 15 min per tape^3,6,7^. Due to the low abundance of many proteins relevant for inflammatory skin diseases in the stratum corneum^1,5^, multiple tapes are often pooled by sequential sonication in the same buffer, concentrating the extract^6,8–10^. This exposes the buffer and extracted proteins therein to a greater cumulative sonication time. Sonication is used to mechanically detach proteins from tapes^1^ and is useful, for example, in extraction protocols involving mass spectrometry as a downstream method of protein characterization, whereby digestion into peptides is often performed before quantification^11,12^. However, we hypothesize that harsh extraction methods, such as prolonged sonication, may be unnecessary in the setting of tape strips, given the easier accessibility to proteins compared to more complex biopsy types. Furthermore, we suspect whether harsh methods may inadvertently degrade proteins, reducing their detection on assays such as the enzyme-linked immunosorbent assay (ELISA) or Meso Scale Discovery (MSD), which require intact protein structure. To that effect, some sonication protocols advise using on/off pulses to avoid overheating samples, although this is not universally followed^5,13,14^.

Few studies have examined the impact of such methodological factors on intact protein extraction from tape strips^15,16^. Indeed, some studies do not describe sonication settings, buffer type, or other protocol components in detail^5,17^, limiting their reproducibility, leaving researchers without clear methodological guidance, and overall highlighting the historically underappreciated importance of these factors in tape strip research. This gap is notable given the increasing adoption of tape stripping in translational and clinical dermatology research^2^.

In this project, we perform a method optimization investigation, describing experimental parameters which can impact results when extracting intact proteins from tape strips. These include extraction buffer type, its volume, and sonication time. Using equivalent input material in all comparisons by cutting tapes, we find that these variables, and the inclusion of appropriate negative controls, are crucial when designing studies. By systematically examining these factors, we provide practical guidance to inform protocol design, thereby improving data quality, comparability, and reproducibility in future studies.

## Methods

All method schematics were created using Adobe Illustrator, version 29.2.1, https://www.adobe.com/dk/products/illustrator/free-trial-download.html.

### Sample collection

The study was conducted in accordance with the guidelines of the Declaration of Helsinki and was approved by the local ethics committee (H-21014505) and the Danish Data Protection Agency (P-2022-80). Oral and written information was provided by the investigator, and informed consent was obtained from 13 healthy controls and 19 CHE patients recruited at the Department of Dermato-venereology at Bispebjerg Hospital, Copenhagen, Denmark. Palmar and dorsal lesional CHE sampling sites were included. Systemic therapy use was an exclusion criterion. Healthy controls were age-, sex-, and sampling location-matched. D-Squame D100 tapes (22 mm diameter, 3.8 cm^2^) (Clinical and Derm) were applied at a pressure of 225 g/cm^2^ for 10 s using the D-Squame pressurizer (Clinical and Derm), before storage at -70 °C until analysis. Sequential tapes were sampled from each subject, and tapes number 13, 15, 17, and in some cases 19, were pooled during protein extraction for each sample, as specified per experiment.

## Protein extraction

Extraction buffers consisted of PBS with or without 0.005% Tween-20 (PBS-T), or T-PER buffer (Thermo Scientific, 78510). Additionally, 25 mM of the protease inhibitor 2-chloroacetamide (CAA) (Thermo Scientific, A15238.0I) was supplemented, unless otherwise stated. Tapes were cut to allow equivalent input material to be distributed among experimental conditions. They were handled with surgical forceps and scissors cleaned with 70% ethanol between samples. Tape quarters (or halves) were placed in round-bottom low protein-binding tubes (Eppendorf, 0030108450) with extraction buffer, then centrifuged at 4 °C and 14,000 g for 30 s, to remove bubbles which may introduce variability during sonication. Sonication in a 0–4 °C ice bath was performed 8 samples at a time, with 50% amplitude and 30-second on/off pulses (Fisherbrand, FB505). All sonication times reported herein refer to the ‘on’ sonication time, meaning that total machine run times are twice the reported duration when accounting for on/off cycles. For sonication exceeding 16 min, ice was replenished in the sonication bath. When pooling tapes, the first tape quarters (or halves) were sonicated, transferred using tweezers to a separate tube for brief centrifugation to retrieve any remaining buffer, then discarded. The subsequent tape quarters/halves were then placed in the same buffer, and the process repeated.

## Protein concentration measurement

Total protein concentration was determined using the Pierce Micro BCA assay (Thermo Scientific, 23235). Following manufacturer instructions, plates were briefly agitated on a plate shaker, then incubated for 2 h at 37 °C, before absorbance reading at 562 nm. Interleukin- (IL-) 1β was measured by ELISA (Invitrogen, 88-7261-88), following manufacturer instructions. The MSD V-PLEX Proinflammatory Panel 1 Human kit (Meso Scale Diagnostics, K15049D) was used in some experiments to measure cytokine concentration at higher sensitivity than ELISA. Manufacturer instructions were followed, with the exception of performing an overnight incubation of undiluted samples at 4 °C, to improve detection.

## Sonication and measurement of recombinant human cytokines

Recombinant human cytokines were reconstituted to their manufacturer-recommended concentrations in either deionized water or ELISA assay diluent, before allocation into round-bottom low protein binding tubes (Eppendorf, 0030108450) and performing either 0–16 min of sonication, at the settings described above. Cytokine concentrations were measured using the corresponding ELISA kits. All kits, including the recombinant cytokine standards, were from Invitrogen as follows: IFNγ (88-7316-88); IL-1β (88-7261-88); IL-4 (88-7046-88); IL-6 (88-7066-88); IL-13 (88-7439-88); IL-17 A (88-7176-88); IL-22 (88-7522-88); IL-23 (88-7237-88).

### Statistical analysis

Non-parametric statistical analyses were performed using GraphPad Prism, version 10.4.0, as specified by experiment. The Mann-Whitney test was used to compare protein extraction between buffers, independently in healthy controls, CHE patients, unused tapes, and buffer alone, as well as to compare healthy controls to CHE patients, independently for each extraction strategy. The Kruskal-Wallis test with Dunn’s multiple comparisons was used to compare BCA background levels at various sonication timepoints, versus the reference of 0 min, independently for each buffer. Similarly, ELISA and MSD cytokine levels at various sonication durations were compared to their respective shortest duration, independently in healthy controls and in CHE patients. This method was also used to compare all buffer volumes to one another. Multiple paired Wilcoxon tests were used to compare cytokine concentrations at 0 versus 16 min of sonication. A *p*-value < 0.05 was considered statistically significant throughout.

## Results

### The T-PER + CAA buffer improves IL-1β extraction over PBS-T + CAA, at the cost of higher BCA assay background

**Fig. 1:**
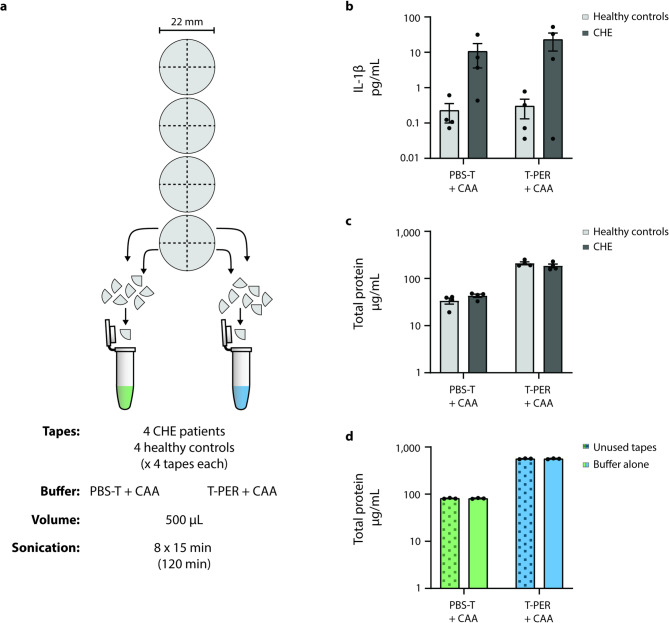
T-PER + CAA extracts more IL-1β than PBS-T + CAA, but produces higher background readings on the BCA assay. (**a**) Schematic representation of the experimental workflow. (**b**) Extraction in T-PER + CAA produced approximately 2-fold higher IL-1β detection compared to PBS-T + CAA. (**c**) The total protein extraction, as measured with the Micro BCA assay, suggests a higher total protein extraction by T-PER + CAA (*p* = 0.056), however, (**d**) a similar pattern was observed when only unused tape strips submerged in buffer, or the buffers alone, were sonicated in the same manner. *n* = 1, with 3 technical replicates. (**a**–**c**) *n* = 4, averages of 3 technical replicates. (**b**–**d**) Multiple Mann-Whitney tests (Holm-Šídák method) comparing protein extraction between buffers, independently for each cohort.

The extraction buffers PBS-T + CAA and T-PER + CAA were compared using tapes from 4 CHE patients and 4 healthy controls (Fig. 1a). Four tapes (number 13, 15, 17, 19) from each subject were cut into quarters, with 8 out of 16 quarters allocated to either buffer to ensure equivalent input material. Similarly to protocols commonly used in the literature, the tape quarters were sequentially sonicated for 15 min, resulting in a cumulative sonication exposure time of 120 min once all 8 quarters were pooled. We found that T-PER + CAA produced an approximately 2-fold higher extraction of IL-1β compared to PBS-T + CAA (Fig. 1b). When measuring the same samples on the highly sensitive MSD assay (Supplementary Fig.S1), 4 out of the 5 detectable cytokines similarly demonstrated improved extraction with T-PER + CAA, with IL-8 being the exception. Total protein extraction, as measured by the BCA assay, also appeared higher with T-PER + CAA in CHE and control tapes (Fig. 1c), however, a similar increase was observed when negative controls (consisting of unused tapes in buffer, or the buffers alone) were sonicated in the same manner (Fig. 1d), suggesting a higher BCA background reading instead. When measuring these negative controls on ELISA, IL-1β was not detected (data not shown).

### Longer exposure of buffers to sonication increases BCA assay background signal, with some volumes more susceptible

**Fig. 2:**
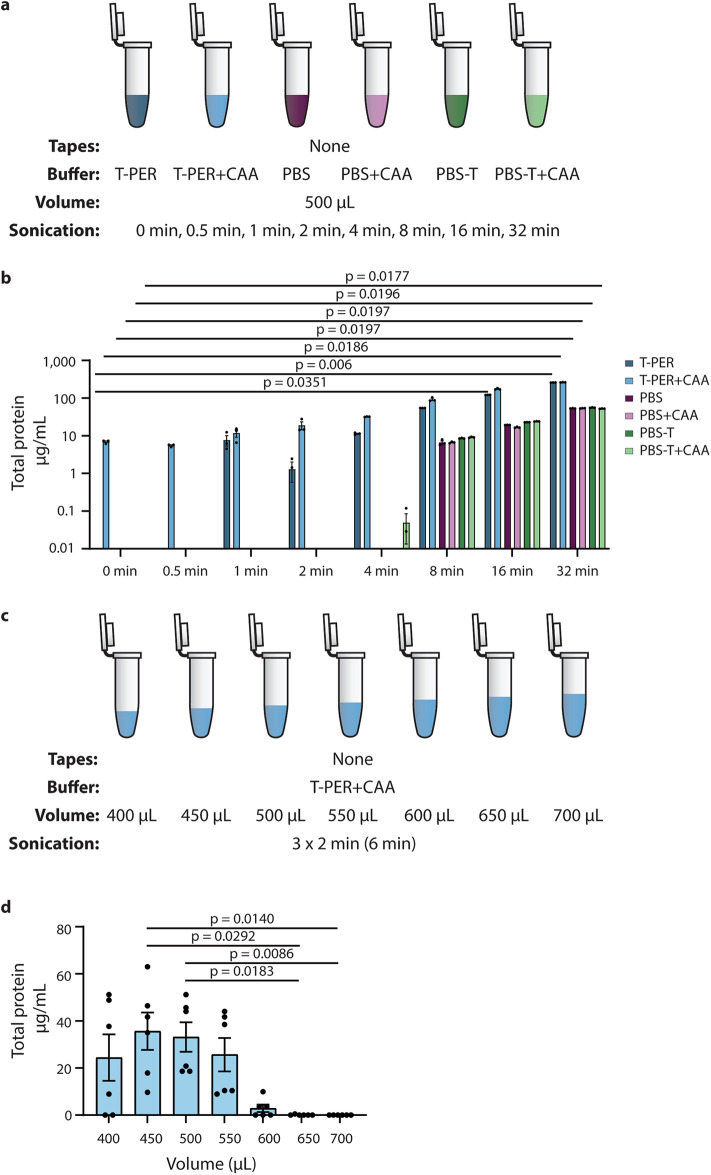
Buffer type, sonication time, and buffer volume impact BCA assay background readings. (**a**) Schematic representation of the experimental workflow in (b). The extraction buffers T-PER, PBS, and PBS-T with or without CAA were sonicated without tapes for various durations. (**b**) Despite the absence of tapes, BCA readings increased with sonication time, particularly with T-PER-based buffers, which had a higher baseline BCA reading than the other buffers at the 500 µL volume used. *n* = 1, with 3 technical replicates for each buffer. Kruskal-Wallis test with Dunn’s multiple comparisons, comparing all durations to 0 min, independently for each buffer. (**c**) Schematic representation of the experimental workflow in (d). (**d**) Buffer volumes ≤ 550 µL produced higher background measurements on the BCA assay. *n* = 2, with 3 technical replicates each. Kruskal-Wallis test with Dunn’s multiple comparisons, comparing all buffer volumes to one another.

To investigate this BCA assay background signal further, we subjected 500 µL of T-PER, PBS, and PBS-T ± CAA to increasing sonication durations (Fig. 2a). We found that the BCA readout increased with sonication time, despite the absence of tapes, revealing an increase in background signal rather than true protein detection (Fig. 2b). Sonicating different volumes of T-PER + CAA, again in the absence of tapes, demonstrated that volume can also impact BCA background levels (Fig. 2c**–**d). Buffer volumes ≤ 550 µL produced higher BCA readings, whereas those ≥ 650 µL produced nearly none, indicating that the comparatively high background from T-PER + CAA with little or no sonication shown in Fig. 2b could be avoided by optimizing the volume. In this buffer volume test, only 6 min of cumulative sonication was used in contrast to the 120 min used previously, mimicking a workflow of pooling three uncut tapes by sequential sonication for 2 min each, with a 5-minute break between each round.

Hence, background signal from T-PER + CAA can be minimized by reducing sonication time and optimizing buffer volume. We next sought to determine whether the use of longer sonication was worthwhile, despite the increased risk of BCA background, if it improved the extraction of proteins of interest.

### Longer sonication does not improve intact protein extraction

**Fig. 3:**
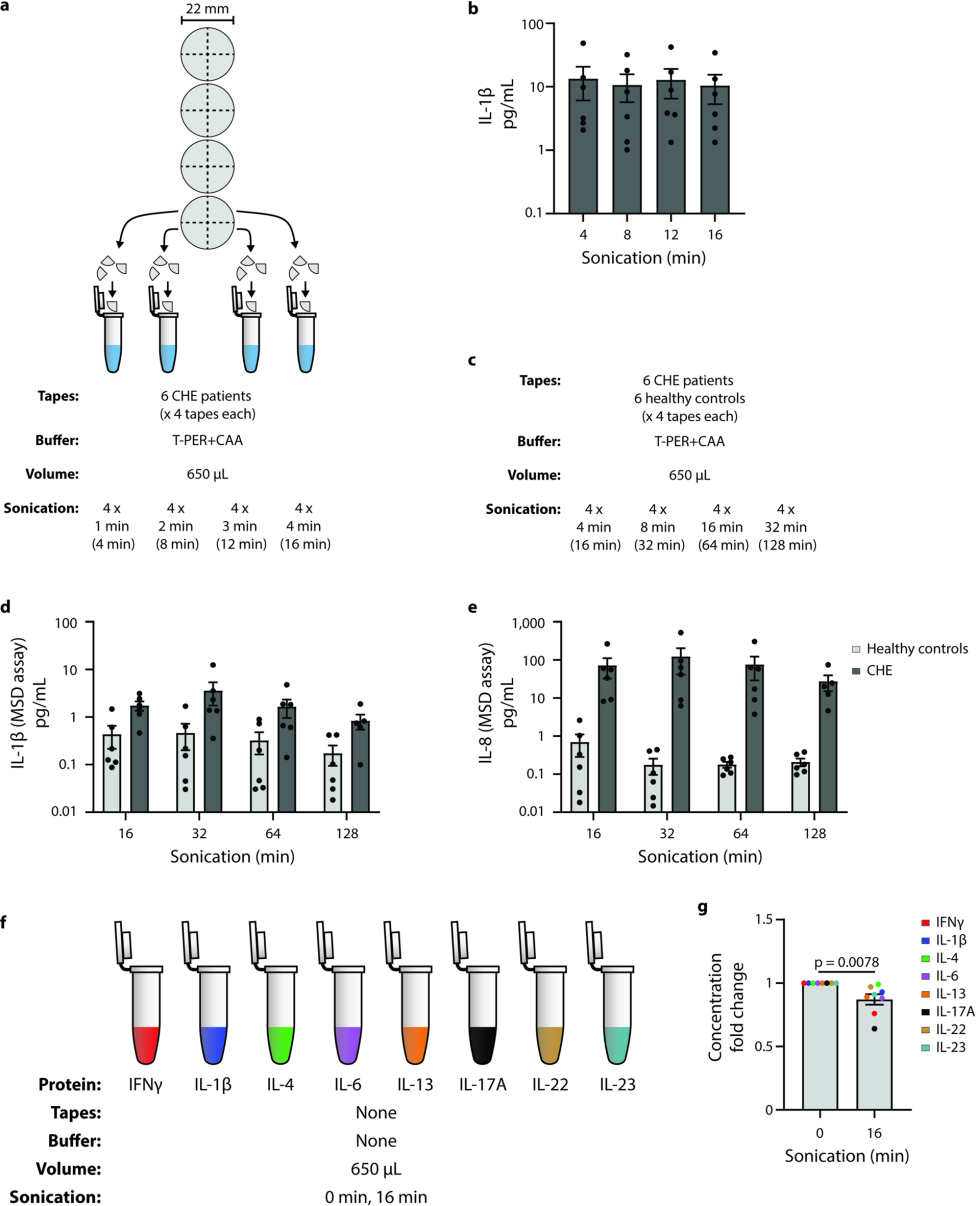
Longer sonication does not improve intact protein extraction. (**a**) Schematic representation of the experimental workflow in (b), whereby 4 tapes were cut into quarters and sequentially sonicated to be pooled, for each of 6 CHE patients. Sonication durations were either 1, 2, 3, or 4 min per round, with each tape equally allocated among the four duration conditions. (**b**) Longer sonication did not improve IL-1β extraction, as measured by ELISA, with the 4 × 1-minute samples having equal or superior concentrations relative to the longer time points. *n* = 6, average of 3 technical replicates each. (**c**) A similar experiment was performed, with both patients and healthy controls, and 4 tapes pooled for each. Sonication durations were increased to 4 rounds of either 4, 8, 16, or 32 min, resulting in cumulative sonication times of 16, 32, 64, and 128 min respectively. (**d**) IL-1β and (**e**) IL-8 levels measured via the MSD assay did not improve with longer sonication. *n* = 5–6, averages of 3 technical replicates. (**b**, **d**, **e**) Kruskal-Wallis test with Dunn’s multiple comparisons. (**f**) In the absence of tapes or extraction buffer, purified recombinant cytokines were measured via ELISA as-is, or first sonicated for 16 min before measurement. (**g**) Cytokines sonicated for 16 min exhibited an average concentration reduction of ~ 15% relative to the unsonicated cytokines. *n* = 1, with 3 technical replicates. Wilcoxon matched-pairs signed rank test (Holm-Šídák method).

When sonicating CHE patient tapes in 650 µL T-PER + CAA, longer sonication did not improve IL-1β extraction, as measured by ELISA (Fig. 3a and b). A similar experimental setup was performed whereby CHE and healthy control tapes were sonicated for 4 rounds of 4, 8, 16, and 32 min each, resulting in cumulative totals of 16, 32, 64, and 128 min (Fig. 3c), and measured on the more sensitive MSD assay. With these prolonged sonication times, only IL-1β and IL-8 were detected, demonstrating no significant change with longer sonication in neither healthy control nor CHE patient tapes (Fig. 3d and e). When subjecting purified recombinant cytokines to the commonly used 16 min of sonication, we observed a ~ 15% average concentration reduction compared to unsonicated counterparts (*p* = 0.0078) (Fig. 3f and g).

With these considerations in mind, we sought to compare two protein extraction strategies to investigate IL-1β levels in CHE patients versus healthy controls.

### Protein extraction from the same tapes using two similar protocols can alter conclusions

**Fig. 4:**
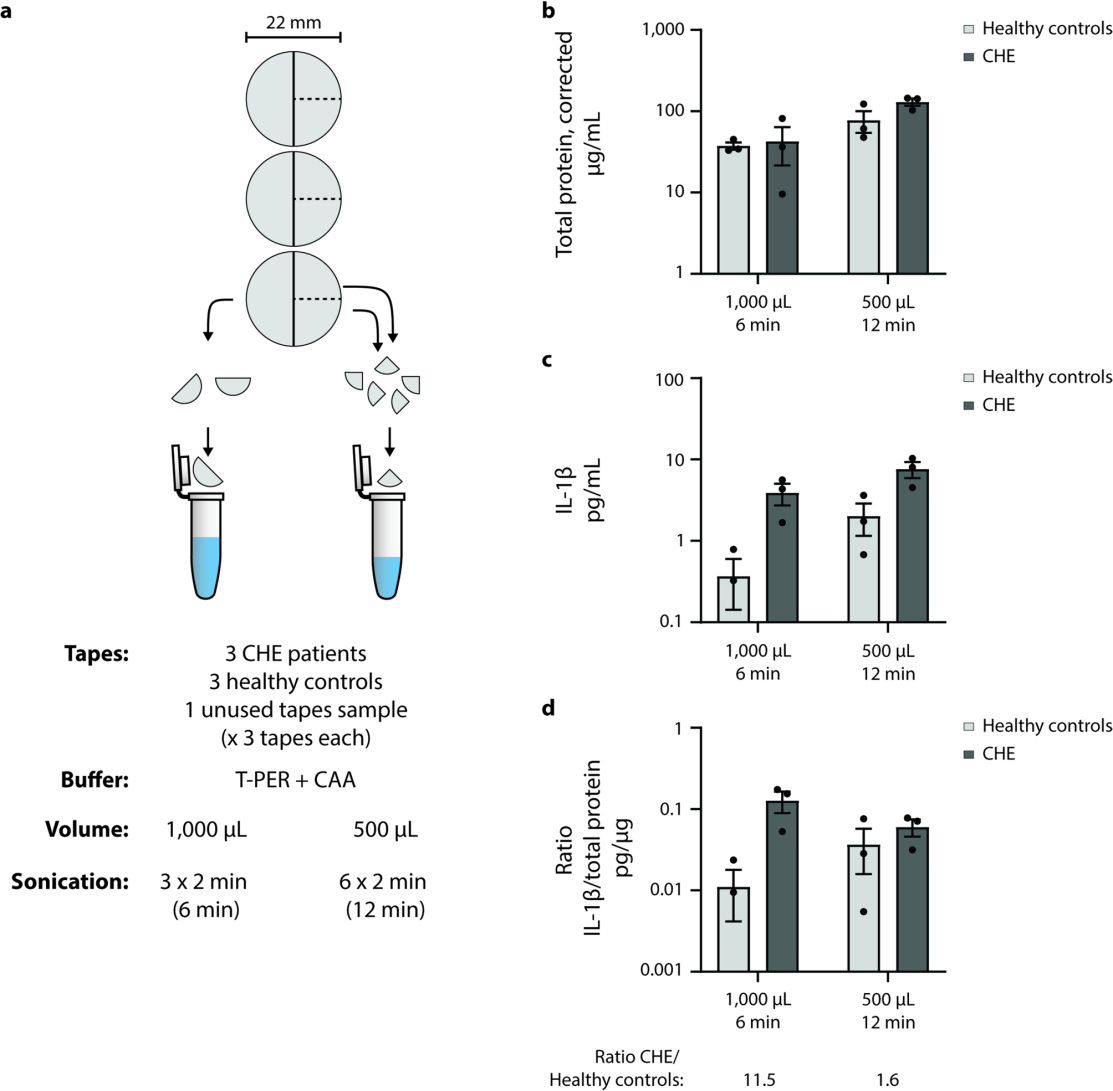
Altering only buffer volume and sonication time in a protein extraction protocol can skew conclusions. (**a**) Schematic representation of the experimental workflow. Three tapes were pooled from each of 3 healthy controls and 3 CHE patients, cutting the tapes to ensure equivalent input material: 3 x ½ tapes in 1,000 µL T-PER + CAA, sonicated for 2 min each, or 6 x ¼ tapes in 500 µL sonicated for 2 min each. Unused tapes treated in the same manner were used as negative controls, with 3 unused tapes pooled together. (**b**) The total protein concentration, corrected for background levels measured on the unused tapes, appears higher in the low volume/high sonication strategy. (**c**) The IL-1β extraction also appears higher in the low volume/high sonication strategy, although these are non-normalized values and reflect the up-concentration of IL-1β in a smaller extract volume. (**d**) The normalization of IL-1β extraction relative to the total protein extraction demonstrates key differences in conclusions to be made with either of these strategies: the high volume/low sonication strategy would indicate that CHE patients have 11.5-fold higher IL-1β levels, while the low volume/high sonication strategy would only suggest 1.6-fold higher levels, as a result of the increased BCA background signal. (**a**–**d**) *n* = 3, averages of 3 technical replicates. Multiple Mann-Whitney tests (Holm-Šídák method).

Three tapes were cut into halves and extracted in 1,000 µL of T-PER + CAA, undergoing 3 sonication rounds of 2 min each for pooling, thus subjecting the buffer to 6 min of cumulative sonication. The other half of each tape was further cut into quarters, which were extracted in 500 µL of T-PER + CAA, undergoing 6 sonication rounds of 2 min each for pooling, thus subjecting the buffer to 12 min of cumulative sonication (Fig. 4a). Unused tape samples were created in the same manner, for use as negative controls. When examining the BCA assay readings, corrected by subtracting the background level measured on the batch-specific negative controls, the low volume/high sonication strategy demonstrated a higher total protein concentration (Fig. 4b), as did the IL-1β concentration measured on ELISA (Fig. 4c). This was expected given the up-concentration of proteins in a smaller volume, with the low volume/high sonication strategy producing 2-fold higher IL-1β levels in the CHE group and 5-fold higher IL-1β levels in the healthy controls (presumably due to greater uncertainty at such low concentrations). However, the ratio of pg IL-1β per µg total extracted protein using the high volume/low sonication strategy suggests 11.5-fold higher IL-1β levels in CHE patients compared to healthy controls, whereas the low volume/high sonication strategy suggests only a 1.6-fold difference (Fig. 4d). Hence, despite the same input material being used in both scenarios, with only buffer volume and sonication time differing, these two strategies provide very different conclusions on this example research question.

## Discussion

The present study highlights the need to consider several methodological variables when designing studies involving intact protein extraction from skin tape strips. Altering extraction buffer type and volume, sonication time, and correction of background signal based on negative controls can greatly impact results in such studies, especially since protein concentrations must be interpreted relative to the total protein extracted from tapes.

There are numerous constituents of extraction buffers that can interfere with the BCA assay used for total protein concentration determination. Reducing agents and chelating agents are known examples: reducing agents can mimic the copper reduction reaction normally accomplished by proteins in the BCA assay, producing false positives^18^, whereas chelating agents can sequester copper ions, preventing them from forming complexes with BCA, creating false negatives^19,20^. Similarly to our own observations, Clausen et al. found that samples containing T-PER exhibited a higher baseline absorbance on the BCA assay, presumably due to the presence of interfering substances producing a false positive signal, drowning out potential biological differences^15^.

Despite this higher background on the total protein concentration assay, T-PER + CAA consistently extracted approximately 2 times higher IL-1β compared to PBS-T + CAA in our experimental setup, using the same tapes and sonication batches, as well as more IL-4, IL-6 and TNFα on the MSD assay. It remains to be elucidated whether the same would be observed for other cytokines relevant in skin research, as IL-8 measured on the MSD assay was found to have improved extraction in the PBS-T + CAA buffer instead. Hendrix et al. have suggested that there is unlikely to exist a single extraction buffer or protocol that universally maximizes recovery of all proteins of interest^16^. Indeed, Metwally et al. further described how the choice of biomarker measurement assay can also preferentially influence the set of proteins detected, particularly for low-abundant biomarkers^21^. All together, these findings highlight the importance of considering extraction buffer candidates based on not only the extraction efficiency for the proteins of interest, but also the compatibility with the total protein concentration determination assay and any other downstream analyses performed. Detailed reporting of these methodological variables is also crucial, as it is evident that they do impact findings.

Beyond the incompatibility of certain buffer constituents, sonication of liquids can in itself produce incompatible substances: the localized high pressure and temperature of cavitation bubbles created during sonication can chemically modify buffer constituents to produce reducing agents or other interfering compounds^22–24^. Indeed, all buffers tested in the absence of tapes, including PBS alone, demonstrated falsely elevated BCA readings when sonicated for 8 min or longer. When considering that tape strip sonication protocols commonly use upwards of 15 min of sonication without on/off cycles, sometimes for several rounds to pool multiple tapes, these results suggest that total protein concentrations can easily be overestimated on the BCA assay. It remains to be tested whether the use of BCA assay compatibility preparation reagents, which can remove background signal-producing substances such as reducing agents, detergents, etc., would be effective in this context. In protocols using buffers with a high potential for background signal even with short sonication time, such additional sample preparation steps may be valuable to consider.

The addition of CAA did not alter BCA background levels to a notable extent, suggesting that it can safely be used as a protease inhibitor. Clausen et al. found that the use of a protease inhibitor in general did not affect protein recovery from tape strips, questioning its necessity in such protocols^15^. Regardless, protease inhibitor candidates should be tested for compatibility before use, as certain constituent reagents, such as dithiothreitol (DTT), are incompatible with the BCA assay and other protein concentration determination assays^25^.

A previous study compared 10- versus 15-minute tape strip sonication protocols, finding no significant difference in total protein extraction as measured by the BCA assay and Squame Scan optical density measurement^15^. However, the use of different tapes in this setting makes direct comparisons between the sonication conditions difficult, given the large discrepancies in material extracted from one tape to the next, even from the same patient^3^. Very short or very long sonication timings were also not assessed, lacking representation of the very high cumulative sonication times commonly used in protocols of 15 min per tape multiplied by several rounds of pooling. Hendrix et al. found that 60 min of sonication decreased intact protein detection on a Luminex-like assay compared to 30 min of sonication, although again using different tapes for each condition, meaning that input material was not equivalent^16^.

Kaleja et al. state that harsh extraction is needed when extracting proteins from tape strips, in order to overcome interactions with the tape adhesive and to lyse cornified cells^12^. However, mass spectrometry was used in this scenario, rather than ELISA or MSD, and our data suggest that harsh extraction is not necessary to extract detectable concentrations of proteins of interest.

Interestingly, background formation on the BCA assay was not linearly related to buffer volume. Instead, intermediate volumes were most susceptible to background formation, while very low or high volumes were not. We speculate that this relates to differences in the magnitude of energy transfer during sonication among different volumes, in relation to the size and shape of the tube used, although this remains to be tested. Hendrix et al. examined protein extraction from tape strips using 1 mL or 1.5 mL of buffer, finding no apparent difference. Although larger than the volumes tested in the present study, and without providing specifications on the tube type, these results are in alignment with our conclusion that volumes ≥ 650 µL were not susceptible to such adverse effects^16^.

Despite this increased propensity for BCA background formation following sonication, we examined whether sonication time should nevertheless be increased for the benefit of improved cytokine extraction, but found no such benefit: IL-1β detection did not increase with longer sonication, and the more sensitive MSD analysis similarly revealed no benefit to cytokine extraction with longer sonication. Protein degradation is a known effect of prolonged sonication^26^, although this effect was not significant in our setup, presumably due to the small sample size. However, sonication of recombinant cytokines for 16 min produced a significant concentration reduction. Whether susceptibility to degradation is cytokine-specific, perhaps in relation to its structure and/or its relative abundance in solution, remains to be investigated. Metwally et al. successfully detected nearly 70% of their 43 proteins assessed on the MSD assay, despite using a cumulative sonication time of 45 min^21^. However, 3 tapes’ worth of material was extracted into 800 µL of PBS-T, meaning that a ‘safe’ buffer type and volume were used with respect to minimizing BCA background potential, reducing the risk of drowning out biomarker signal during normalization. It can be speculated whether the pooling of 3 tapes worth of material created a sufficient concentration of the biomarkers in solution to create a degree of protection from sonication-induced degradation. These precise conditions were not tested in the current setup, but remain an interesting question for future exploration. Our findings on the MSD assay indicate that cumulative sonication times of 16–128 min prevented detection of IFNγ, IL-2, IL-4, IL-6, IL-10, IL-12p70, IL-13 and TNFα when only 1 tape’s worth of material was present in each sample, whereas having 2 tapes’ worth of material still allowed some detection of IL-1β, IL-4, IL-6, IL-8, and TNFα with 120 min of sonication. Cytokine-specific differences in temperature stability have already been described^27,28^, however, this remains to be examined further in relation to sonication-mediated degradation, the implication being that longer sonication may allow preferential detection of more ‘resilient’ cytokines, presenting another argument for its minimization. Beyond the risk of protein degradation, sonication can alter protein residues by modifying amino acid side groups or breaking chains, potentially affecting detectability on immunoassays relying on intact protein structure^26^. Therefore, considering the results of the present study and above implications, we conclude that shorter sonication is preferable, given the lower risk of BCA assay background formation, and at least equally effective intact protein extraction, if not greater.

Beyond sonication time, the observed discrepancy in conclusions regarding IL-1β levels in CHE patients compared to healthy controls illustrates the importance of buffer volume as well. Given the large variability in material collected on each tape strip^3^, proteins of interest must be normalized to total protein on the tape. It is therefore crucial to minimize BCA assay background readings, as artificially elevated total protein concentrations used for normalization can skew the final interpretation of results. In addition to optimizing the buffer and sonication variables described above, this issue of BCA background can be mitigated with the inclusion of a negative control in each sonication batch, given the evident difficulty in controlling all variables which can potentially impact extraction efficiency and background formation during sonication. Kaleja et al. have previously made similar recommendations, suggesting the use of blank tapes to help account for potential contamination arising from tape handling during mass spectrometry sample preparation^12^. Regardless of the precise experimental setup in question, the use of negative controls is an important strategy for correction of background levels, reducing batch effects and improving reproducibility.

A key limitation of this study was the assumption that tape cutting allows for equivalent distribution of input material among experimental conditions. As described by others^9^, stratum corneum collection is not perfectly uniform across the surface of a tape, meaning that the input material was likely not identical across all conditions. Nevertheless, it has been assumed herein that that the heterogeneity within a single tape is lower than that between separate tapes, and that cutting tapes is thus an important step in this study to allow reasonable comparisons between extraction conditions. Furthermore, the present study used tape strips 13, 15, 17, and 19, since the more superficial tapes that are typical for protein assessments (tapes 4–8)^7,8,11,13,21^ were reserved for use in a separate study. While the total number of tapes collected varies depending on objectives, most studies include between 10 and 30 tapes, typically discarding the first two and using the remainder for various analyses^2^. Based on the findings by Clausen et al. describing cytokine and total protein concentrations across the depth of the stratum corneum, our total protein levels measured by the BCA assay on deeper tape layers are likely representative of the more superficial tapes, as are our biomarker concentrations in the CHE patient tapes, which were collected from lesional skin. However, some biomarker concentrations in the healthy controls may be under-representative, as Clausen et al. found a reduced concentration of certain cytokines in this cohort with deeper tape progression^3^. Finally, this study did not include a direct positive control in the form of tape strips spiked with known concentrations of cytokines. While such an approach could facilitate comparison of apparent recovery across extraction conditions (subject to sonication batch effects), spiked cytokines would not fully recapitulate the behavior of endogenous stratum corneum–associated proteins.

The BCA assay is not the only method of assessing total protein concentration from tape strips for biomarker normalization: Squame Scan optical density measurement has also been used, whereby absorption can be measured directly from the tapes at 850 nm before and after extraction, and the difference used as an indirect determinant of the concentration of protein retrieved from the tape^3^. However, this method only scans approximately half of the surface of the tape, meaning that the uneven collection of stratum corneum across the surface of a tape may not be fully accounted for^2,8,14^. It remains to be explored whether other normalization methods beyond total protein concentration could be more suitable in skin tape strip research, avoiding many of the issues presented in this study. It has been suggested that total protein levels in the skin are positively correlated with eczema-related erythema, and can therefore be used as a proxy for the assessment of erythema severity in skin of colour, where visual assessment can underestimate results^8^. Hence, in such settings with biologically valid differences in total protein concentration between patients and healthy controls, other methods of normalization may be more suitable, such as the use of a housekeeping protein. This remains to be investigated further, with housekeeping protein candidates being potentially identifiable using public proteomics databases.

Nevertheless, the present study provides concrete steps which can be taken to optimize the extraction of intact proteins of interest from skin tape strips, ensuring minimal background on the BCA assay commonly used for normalization, and reducing the potential for degradation of susceptible proteins of interest below detection limits on assays such as ELISA or MSD.

## Conclusions

When investigating protein biomarkers from skin tape strips, the limited amount of tissue collected inherently restricts detection of low-abundance proteins. Moreover, it is imperative to normalize biomarker levels to the total amount of protein extracted, which can vary considerably between tapes. Consequently, protein extraction protocols for MSD and ELISA-type assays should aim to maximize recovery of target proteins while minimizing background signals in the total protein assay. While T-PER buffer enhances biomarker extraction, it also increases background readings of total protein on the BCA assay, particularly when using a low buffer volume and long sonication times. Hence, in this study, we highlight the importance of considering extraction buffer type, its volume, and sonication time when designing experiments involving extraction of intact proteins from tape strips. Furthermore, the inclusion of unused tapes as negative controls for each sonication batch is highly recommended, to account for batch-specific variation.

## Supplementary Information

Below is the link to the electronic supplementary material.

Supplementary Material 1

## Data Availability

The data that support the findings of this study are available from the corresponding authors upon reasonable request.
