# Supplementary material for: Optimization of protein extraction from skin tape strips for biomarker assessment
Source: Sci Rep. 2026 Apr 11;16:17221. doi: 10.1038/s41598-026-44234-9 (PMC13234127; doi:10.1038/s41598-026-44234-9)
Supplement: Supplementary file 1 — Supplementary Material 1 [file 41598_2026_44234_MOESM1_ESM.pdf]

Optimization of protein extraction from skin tape strips for biomarker assessment

Tiana Stanisic[1,2]*, Caroline Meyer Olesen[3,4], Maria Oberländer Christensen[3], Beatrice Dyring-Andersen[1,2] Marianne Bengtson Løvendorf[1,5], Jop Vreeken[6], Florentine de Boer[6], Sanja Kezic[6] & Martin Kongsbak-Wismann[1]*

[1] LEO Foundation Skin Immunology Research Center, Faculty of Health and Medical Sciences, University of Copenhagen, Copenhagen, Denmark
[2] Department of Dermatology, Zealand University Hospital, Roskilde, Denmark
[3] Department of Dermato-venereology, Bispebjerg Hospital, Copenhagen, Denmark
[4] LEO Pharma A/S, Ballerup, Denmark
[5] Department of Dermatology and Allergy, Herlev and Gentofte Hospital, Copenhagen University Hospitals, Copenhagen, Denmark
[6] Amsterdam Public Health Research Institute, Department of Public and Occupational Health, Amsterdam University Medical Center, University of Amsterdam, Amsterdam, The Netherlands

Corresponding authors
*Tiana Stanisic tiana.stanisic@sund.ku.dk
*Martin Kongsbak-Wismann mkong@sund.ku.dk

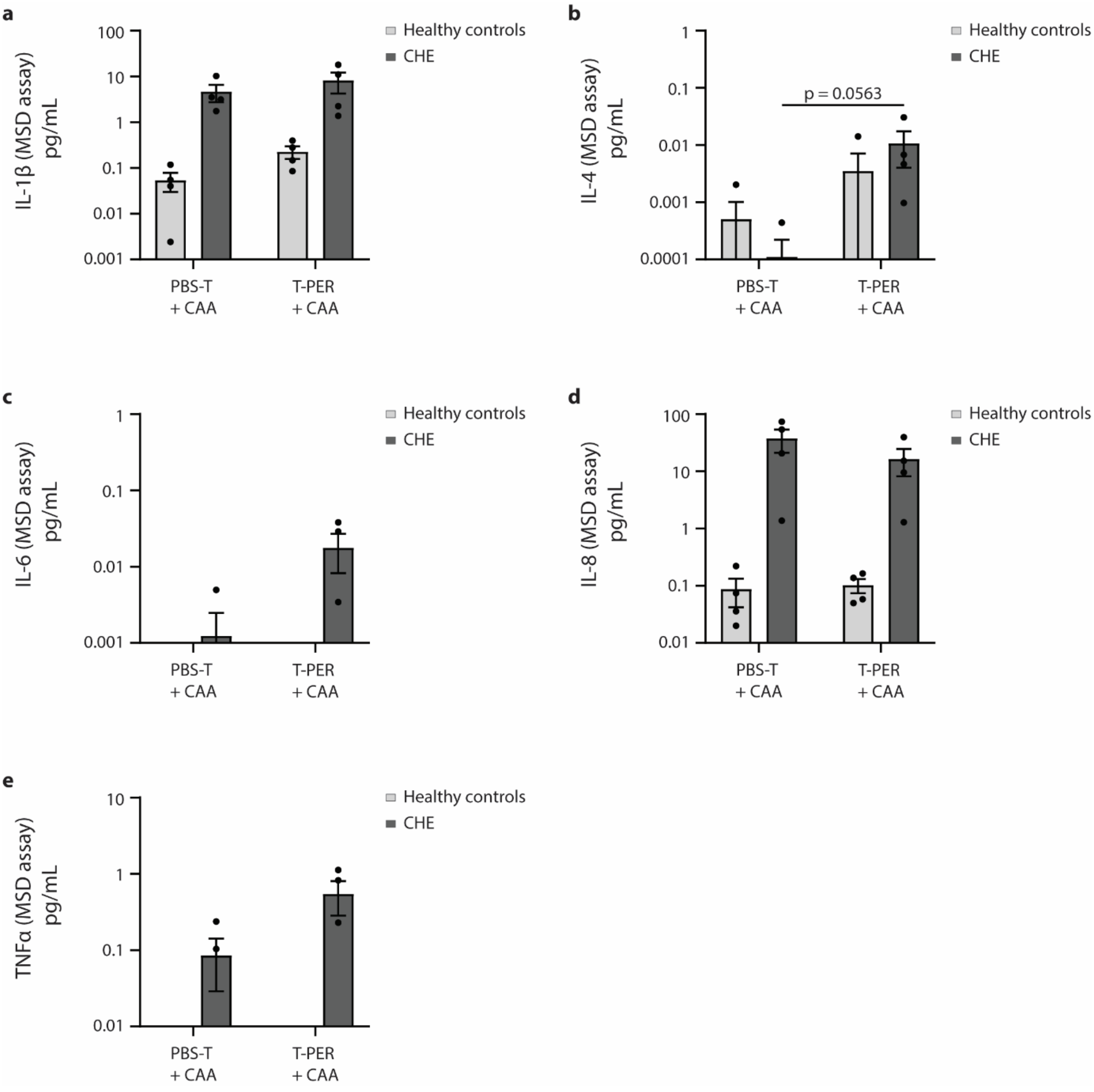

Supplementary Figure S1. Buffer extraction efficiency of cytokines detectable on the MSD assay. Tape strips from 4 CHE patients and 4 healthy controls were processed as described in Fig. 1a, before biomarker measurement using the MSD assay. (**a**) IL-1β levels demonstrate similar results to those observed on ELISA (Figure 1b), with a tendency for T-PER + CAA to extract higher levels from both CHE patient tapes and healthy controls. (**b**) IL-4 and (**c**) IL-6 were largely below the detection threshold in samples extracted in PBS-T + CAA and in healthy controls, whereas all CHE patient samples extracted in T-PER + CAA were detectable. A trend of higher extraction by T-PER + CAA was observed for IL-4. (**d**) IL-8 levels were conversely higher in CHE samples extracted in PBS-T + CAA, whereas control samples exhibited no difference between buffer types. (**e**) TNFα was only detectable in CHE patient samples, and to an approximately 4 times higher degree when T-PER + CAA buffer was used. (**a – e**) n = 4, averages of 3 technical replicates. Multiple Mann-Whitney tests (Holm-Šídák method).
